# Ligand-based prediction of hERG-mediated cardiotoxicity based on the integration of different machine learning techniques

**DOI:** 10.3389/fphar.2022.951083

**Published:** 2022-09-05

**Authors:** Pietro Delre, Giovanna J. Lavado, Giuseppe Lamanna, Michele Saviano, Alessandra Roncaglioni, Emilio Benfenati, Giuseppe Felice Mangiatordi, Domenico Gadaleta

**Affiliations:** ^1^ CNR—Institute of Crystallography, Bari, Italy; ^2^ Chemistry Department, University of Bari “Aldo Moro”, Bari, Italy; ^3^ Laboratory of Environmental Chemistry and Toxicology, Department of Environmental Health Sciences, Istituto di Ricerche Farmacologiche Mario Negri IRCCS, Milan, Italy; ^4^ CNR-Institute of Crystallography, Caserta, Italy

**Keywords:** hERG, cardiotoxicity, QSAR, ligand-based, consensus modeling

## Abstract

Drug-induced cardiotoxicity is a common side effect of drugs in clinical use or under postmarket surveillance and is commonly due to off-target interactions with the cardiac human-ether-a-go-go-related (hERG) potassium channel. Therefore, prioritizing drug candidates based on their hERG blocking potential is a mandatory step in the early preclinical stage of a drug discovery program. Herein, we trained and properly validated 30 ligand-based classifiers of hERG-related cardiotoxicity based on 7,963 curated compounds extracted by the freely accessible repository ChEMBL (version 25). Different machine learning algorithms were tested, namely, random forest, K-nearest neighbors, gradient boosting, extreme gradient boosting, multilayer perceptron, and support vector machine. The application of 1) the best practices for data curation, 2) the feature selection method VSURF, and 3) the synthetic minority oversampling technique (SMOTE) to properly handle the unbalanced data, allowed for the development of highly predictive models (BA_MAX_ = 0.91, AUC_MAX_ = 0.95). Remarkably, the undertaken temporal validation approach not only supported the predictivity of the herein presented classifiers but also suggested their ability to outperform those models commonly used in the literature. From a more methodological point of view, the study put forward a new computational workflow, freely available in the GitHub repository (https://github.com/PDelre93/hERG-QSAR), as valuable for building highly predictive models of *hERG*-mediated cardiotoxicity.

## Introduction

### Background

Cardiotoxicity is a common side effect of drugs, and one of the causes for it is the off-target interaction with different voltage-gated ion channels expressed in the heart (Ferdinandy et al., 2019). Among the others, the human ether-a-go-go related (hERG) channel has received increasing attention over the past few decades as several drugs have been restricted in their use or withdrawn because of their ability to block this channel by interacting with a hydrophobic pocket called central cavity (Kalyaanamoorthy and Barakat, 2018; Butler et al., 2020). Remarkably, a drug-induced hERG blockade can be responsible for potentially lethal cardiac arrhythmias in the form of the so-called long-QT syndrome (LQTS) (Priest et al., 2008; Danker and Möller, 2014). Since drugs belonging to very different chemical classes were proved to cause this severe side effect, an early evaluation of hERG blockade has become a necessary step during the development of drug discovery (DD) programs (Kalyaanamoorthy and Barakat, 2018; Ferdinandy et al., 2019; Cavalluzzi et al., 2020). Meaningful examples are represented by terfenadine (Kamiya et al., 2008; Tanaka et al., 2014), astemizole (Zhou et al., 1999), cisapride (Walker et al., 1999; Kamiya et al., 2008), and ziprasidone (Su et al., 2006). As a matter of fact, submission to regulatory reviews requires a preclinical assessment of hERG blockage activities, as clearly indicated by the guidelines defined at the International Conference on Harmonization of Technical Requirements for the Registration of Pharmaceuticals for Human Use (ICH) (EMA, 2005; FDA, 2005).

### *In silico* evaluation of hERG blockade

In this context, to avoid hERG liability during the DD process and, therefore, prioritize safe drug candidates at the early preclinical stage, the employment of *in silico* tools is highly desirable since *in vitro* (e.g., fluorescence-based assays, electrophysiology measurements, rubidium-flux assays, radioligand binding assays) and *in vivo* experiments are much more laborious, time-consuming, and expensive (Priest et al., 2008; Jing et al., 2015). Accordingly, several *in silico* tools have been developed in the last few years using both ligand- and structure-based approaches (Jing et al., 2015; Villoutreix and Taboureau, 2015; Kalyaanamoorthy and Barakat, 2018; Creanza et al., 2021). In the absence of an atomic-resolution hERG structure, the attention of both academia and industry has been mainly focused on the development of ligand-based classifiers (e.g., pharmacophore models, quantitative structure–activity relationship (QSAR) approaches) (Jing et al., 2015; Villoutreix and Taboureau, 2015; Slavov et al., 2017; Sun et al., 2017; Kalyaanamoorthy and Barakat, 2018). The interested reader is referred to references (Jing et al., 2015; Villoutreix and Taboureau, 2015; Kalyaanamoorthy and Barakat, 2018) for comprehensive reviews on this topic. However, despite providing good performances, many ligand-based models developed so far suffer from critical limitations. Most of them, in fact, were built from a limited number of congeneric analogs (Chavan et al., 2016; Gobbi et al., 2016; Wang et al., 2016; Zhang et al., 2016; Munawar et al., 2018; Liu et al., 2020), and for this reason, their applicability domain (AD) (Gadaleta et al., 2016) is too restricted for a real-life application, since hERG blockers are characterized by high structural diversity. Recent articles published by Cai et al. (2019), Ryu et al. (2020), and Karim et al. (2021) reported classifiers trained on more than 7,000 compounds, hence encompassing a broad AD. The authors used deep learning techniques and IC_50_ = 10 μM as the toxicity threshold to discern hERG blockers from nonblockers. As a result, these models ensured performances better than those achieved using more traditional machine learning (ML) approaches (AUC_MAX_ = 0.88, 0.90, and 0.91, respectively). However, the real-life application of such classifiers might be questionable as substances considered of critical concern in DD programs are responsible for IC_50_ lower than 1 μM (rather than 10 μM) (Zolotoy et al., 2003; Katchman et al., 2006; Kim et al., 2008; Hong et al., 2013). In this regard, Krishna et al. (Krishna et al., 2022) recently developed QSAR models based on Tox21 quantitative high throughput screening (qHTS) thallium flux assay and ChEMBL (v27) data using IC_50_ = 10 μM as the toxicity threshold to discern hERG blockers from nonblockers. Different ML methods and consensus modeling were evaluated. The best models were ultimately integrated into a consensus that improved the performance of the single best ones (BA = 0.791 on the external set).

### Objectives

Building on these pieces of evidence and background, in this study we report several ligand-based models developed starting from 7,963 curated compounds (hERG-DB) and extracted from the ChEMBL (Gaulton et al., 2012) version (v) 25, employing six classification algorithms, random forest (RF) (Breiman, 2001), K-nearest neighbors (KNN) (Altman, 1992), gradient boosting (GB) (Friedman, 2001), extreme gradient boosting (XGB) (Chen and Guestrin, 2016), multilayer perceptron (MLP) (Haykin, 1994), and support vector machine (SVM) (Vapnik, 1963), and as toxicity thresholds both IC_50_ = 1 and IC_50_ = 10 μM. Notably, the models returning the best performances were challenged on a supplementary external set (ES) consisting of molecules recovered from the new CHEMBL (Gaulton et al., 2012) update (v28) and therefore not included in hERG-DB (see section “*Materials and methods*” for methodological details). This procedure, also known as time-split cross-validation or temporal validation and involving data published a posteriori, is today considered of utmost importance to test the real-life applicability of the developed classifiers (Sheridan, 2013; Bosc et al., 2019). Last but not least, six ligand-based hERG models available in the literature and named DeepHIT (Ryu et al., 2020), CardioTox (Karim et al., 2021), Cardprep (Lee et al., 2019), ADMETlab (Xiong et al., 2021), and OCHEM consensus models I and II (Li et al., 2017) were applied on the ES to compare their performance with that returned by our top-performing model. The obtained results put forward the herein presented computational workflow as valuable for a robust ligand-based prediction of hERG-related cardiotoxicity. This study can be useful also considering that cardiovascular diseases are ranked high in the top causes of sudden death (Onakpoya et al., 2016).

## Materials and methods

### Dataset preparation

A total of 17,952 activity entries were extracted from ChEMBL (Gaulton et al., 2012) v25 according to the Target ID (ChEMBL240) assigned to the hERG channel. To ensure data validity, the database was mined retaining only those entries matching the following criteria already suggested in the literature: 1) annotated exclusively with IC_50_ (11,144 entries) measures, 2) referring to assays conducted on human targets (“target_organism” = “*Homo sapiens*”), 3) marked as direct binding (“assay_type” = “B”), and 4) free of warnings in the “data_validity_comment” field (Alberga et al., 2019; Bosc et al., 2019; Creanza et al., 2021). SMILES were curated using a semiautomated in-house procedure described by Gadaleta et al. (2018a). Such a process allows for removing organometallic and inorganic compounds, chemicals characterized by unusual elements and mixtures, neutralizing salts, and removing stereochemistry. The neutralized SMILES were converted to a standardized QSAR-ready format using OpenBabel (O’Boyle et al., 2011) implemented in the KNIME Analytics Platform (Berthold et al., 2008) to generate canonical SMILES. Each IC_50_ value was converted from molar concentration (M) to pIC_50_ (–log IC_50_), and compounds devoid of any pIC_50_ value but already marked as not active in the ChEMBL repository were also considered. In the last step, duplicates were aggregated in unique entries and the standard deviation (σ) related to the pIC_50_ values was computed. Also, 15 compounds were considered outliers (σ > 2) while for all of the others, the average pIC_50_ value was considered. In such a way, the curated dataset consists of 7,963 chemicals (hereinafter referred to as hERG-DB) and the corresponding experimental value. Consistent with the literature (Jing et al., 2015; Zolotoy et al., 2003), hERG-DB includes hERG blockers (ACT) having an IC_50_ ≤ 1 µM (pIC_50_ ≤ 6), compounds showing a moderate hERG blocker potential with an IC_50_ ranging from 1 to 10 µM (6 < pIC_50_ ≤ 5), and, finally, hERG nonblockers (INA) having IC_50_ values >10 µM (pIC_50_ > 5). For this reason, in this work, we have developed two sets of binary models differing for the considered toxicity threshold (pIC_50_ = 6 or pIC_50_ = 5). Finally, we downloaded the recent version of ChEMBL (Gaulton et al., 2012) (version 28) to extract possible compounds not present in our hERG-DB, following the same data curation process described above. Thus, an external (ES) dataset of 792 chemicals was assembled and curated with the same procedure described above and then used to challenge the real-life predictivity of the top-performing classifiers.

### Dataset division

We split hERG-DB into a training set (TS) and a validation set (VS) following a rational approach. Notably, the RDkit Picker Diversity node (Landrum et al., 2022) was employed separately on the two classes (i.e., ACT and INA) resulting from the considered toxicity thresholds to generate the Morgan fingerprints (Rogers and Hahn, 2010) for each SMILES, and 80% of the most diverse molecules was then picked based on the Tanimoto distance (Willett et al., 1998). In doing so, the resulting TS included 80% of the total molecules (6,371); the remaining 20% (1592) constituted the VS. Table 1 summarizes the final composition of TS, VS, and ES, indicating the number of ACT and INA for each of the two selected toxicity thresholds. Note that such a procedure allowed us to keep the INA/ACT ratio fixed in each subdivision. Interestingly, although not prepared by a rational strategy, the ES presented an INA/ACT ratio in line with those of TS and VS.

**TABLE 1:** Partitioning schemes before (top) and after the application of the AD at each considered toxicity threshold (bottom). For *hERG-DB*, the number of active and inactive chemicals and the related class distribution is reported for the training set (TS), validation set (VS), and external set (ES) and at each considered toxicity threshold. Notably, the total number of chemicals (#), the number of *hERG* blockers (ACT) and *hERG* non-blocker (INA) chemicals, as well as the ratio between nonblockers and blockers are shown.

Dataset	Toxicity threshold (pIC_50_)							
	6				5			
	#	INA	ACT	INA:ACT	#	INA	ACT	INA:ACT
Starting composition								
TS	6371	5388	983	05:01	6371	3295	3076	01:01
VS	1592	1346	246	05:01	1592	821	771	01:01
ES	792	676	116	05:01	792	365	427	01:01
Applicability domain (AD)								
VS	1583	1338	245	05:01	1579	810	769	01:01
ES	754	642	112	05:01	754	350	404	01:01

**FIGURE 1:**
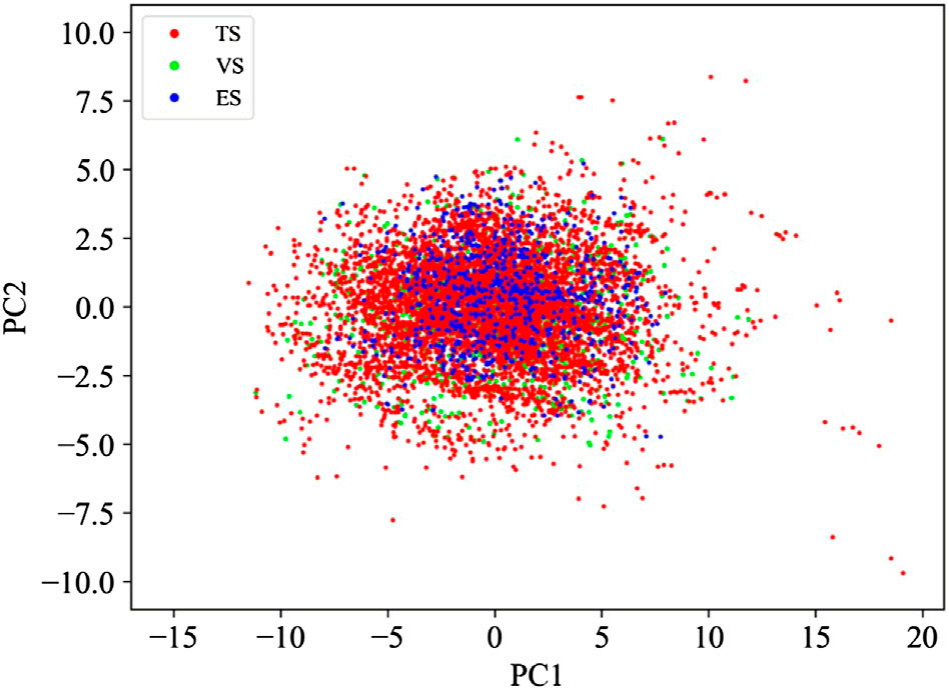
PCA based on the physicochemical properties returned by the compounds belonging to TS, VS, and ES.

The splitting procedure was challenged by performing a principal component analysis (PCA) (Jolliffe and Cadima, 2016) based on the physicochemical properties of the molecules calculated by the molecular properties KNIME node based on the CDK toolkit (Steinbeck et al., 2003; CDK, 2022. Available at: https://cdk.github.io/). The score plot of the first two principal components, able to capture more than 90% of the data variance, confirms that the described procedure ensured a uniform distribution of the compounds in the TS and VS throughout the model space (Figure 1). In addition, Figure 1 includes the PCA of the ES. It is worth noting that, although not derived by a splitting approach, this covers a chemical space similar to those covered by both TS and VS.

### Development of statistically based models

#### Descriptors calculation

We used DRAGON v7.0.4 (Kode, 2017) as a software program to compute the 2D-descriptors of each chemical belonging to the datasets. Descriptors having missing values or constant/near-constant variables (i.e., standard deviation < 0.01) were removed along with those having an absolute pair correlation higher than 95% with other variables. Thus, we finally considered 1,070 descriptors. The obtained values were scaled with a standard normalization (i.e., mean equal to zero and standard deviation equal to 1). Models were built using both the entire pool of descriptors and a reduced set selected by the R package VSURF, an RF algorithm working in three steps to detect variables related to the activity and eliminate those redundant or irrelevant (Genuer et al., 2010). Following this protocol, we finally selected 79 (pIC_50_ threshold = 6) and 86 (pIC_50_ threshold = 5) descriptors (Supplementary Table S1). The feature selection was based on the TS only to remove any putative artifact in the model selection.

#### Model development and validation

For each partitioning scheme reported in Table 1, we used six classification algorithms: RF (Breiman, 2001), KNN (Altman, 1992), GB (Friedman, 2001), XGB (Chen and Guestrin, 2016), MLP (Haykin, 1994), and SVM (Vapnik, 1963). The TS is characterized by an INA: ACT ratio equal to 5:1 when the pIC_50_ toxicity threshold is 6; hence, it is strongly unbalanced. This could favor the convergence of algorithms trained on the majority class, neglecting classes with fewer samples (Zhang et al., 2018). For this reason, in addition to the models developed using this TS, additional models were developed, artificially altering the original TS using the Synthetic minority oversampling technique (SMOTE) to balance the number of blockers and nonblocker samples (Chawla et al., 2002). Such an approach, based on the KNN algorithm and operating in the “feature space”, oversampled the minority class by creating and introducing new synthetic samples until a ratio of INA: ACT of 1:1 is reached. As for the VSURF procedure, the SMOTE was applied only to the TS. VS, indeed, was kept unbalanced to properly evaluate the capability of the classifiers to predict the real distribution of data. In all of the cases, to find the optimal algorithm setting for training the final model, the parameter selection was based on hyperparameter tuning and 5-fold cross-validation (CV) performance (Refaeilzadeh et al., 2009). To do this, we performed a grid search (LaValle et al., 2004), except for SVM and XGB, where we used Bayesian optimization to reduce the computational cost (Snoek et al., 2012). The optimal parameters for each algorithm, selected based on the best metrics in 5-fold CV, are shown in Supplementary Table S2. It is worth noting that RF models, trained on the original unbalanced TS, combine an equal size data sampling for both thresholds pIC_50_ = 5 and pIC_50_ = 6. This technique is also known as balanced random forest (BRF). After parameter setup and model training, top-performing models were selected based on external performance on the VS and then applied to the ES that represented the ultimate proof for real-life validation of the models. Finally, to possibly improve predictions provided by single best models, consensus modeling was applied. In particular, a compound was assigned to a category based on a straightforward majority voting approach, i.e., only when the top-performing models, selected based on the computed balanced accuracy (BA) and area under the curve (AUC) values, generated concordant predictions.

#### Applicability domain

To increase the confidence in the model's prediction, we defined the applicability domain (AD), namely, the chemical space from which the classifiers are derived and, therefore, where a prediction can be considered trustworthy (Roy et al., 2015; Gadaleta et al., 2016; Kar et al., 2018). To define the AD, we used the Enalos Domain—Leverages node for KNIME (Afantitis et al., 2008; Melagraki et al., 2009). This approach allows for the calculation of the leverage (h) for each chemical and defines a threshold that works as an upper bound limit. Compounds with leverage values of h > 3p/n, where p is the number of descriptors and n is the number of molecules, are considered chemically different from the TS compounds (Tropsha et al., 2003; Afantitis et al., 2008; Melagraki et al., 2009). Thus, 9 (threshold pIC_50_ = 6) and 13 (threshold pIC_50_ = 5) were discarded from VS, whereas 38 compounds were excluded from ES for both the considered activity thresholds. Table 1 reports the composition of both VS and ES after applying the AD-based filter.

### Performance evaluation

The performance of the classification models was evaluated using Coopers statistics, i.e., balanced accuracy (BA), sensitivity (SE), and specificity (SP), computed as follows:

$$SE=\;\frac{TP}{TP+FN}$$

$$SP=\;\frac{TN}{TN+FP}$$

$$BA=\;\frac{SE+SP}{2}$$

where true positives (TPs), and true negatives (TNs) are, respectively, the positive and negative samples correctly classified by the models and false negatives (FNs) and false positives (FPs) are the misclassified positive and negative samples, respectively (Ting, 2017). Another used metric was the Matthews correlation coefficient (MCC). MCC indicates the quality of binary classification and is generally recognized as a reliable metric, although it deteriorates seriously when the TS is imbalanced (Zhu, 2020). MCC ranges between −1 and +1. A value of +1 means a perfect classification, 0 indicates a random classification, and −1 is a complete misclassification (Zhu, 2020).

$$MCC=\;\frac{TP*TN-FP*FN}{\sqrt{\left(TP+FP\right)\left(TP+FN\right)\left(TN+FP\right)\left(TN+FN\right)}}$$

Finally, the AUC, namely, the area under the receiver operating characteristic (ROC) curve, was also computed to estimate the predictive accuracy of the models. Notice that the AUC, ranging from 0 (miss-classifiers) to 1 (ideal-classifiers), reflects the probability of positive compounds being ranked earlier than decoy compounds (Fawcett, 2006). This quality metric was computed for each developed model based on the output scores associated with each prediction returned during the validation procedure and estimating the probability that a given compound is an hERG blocker.

## Results and discussion

**FIGURE 2:**
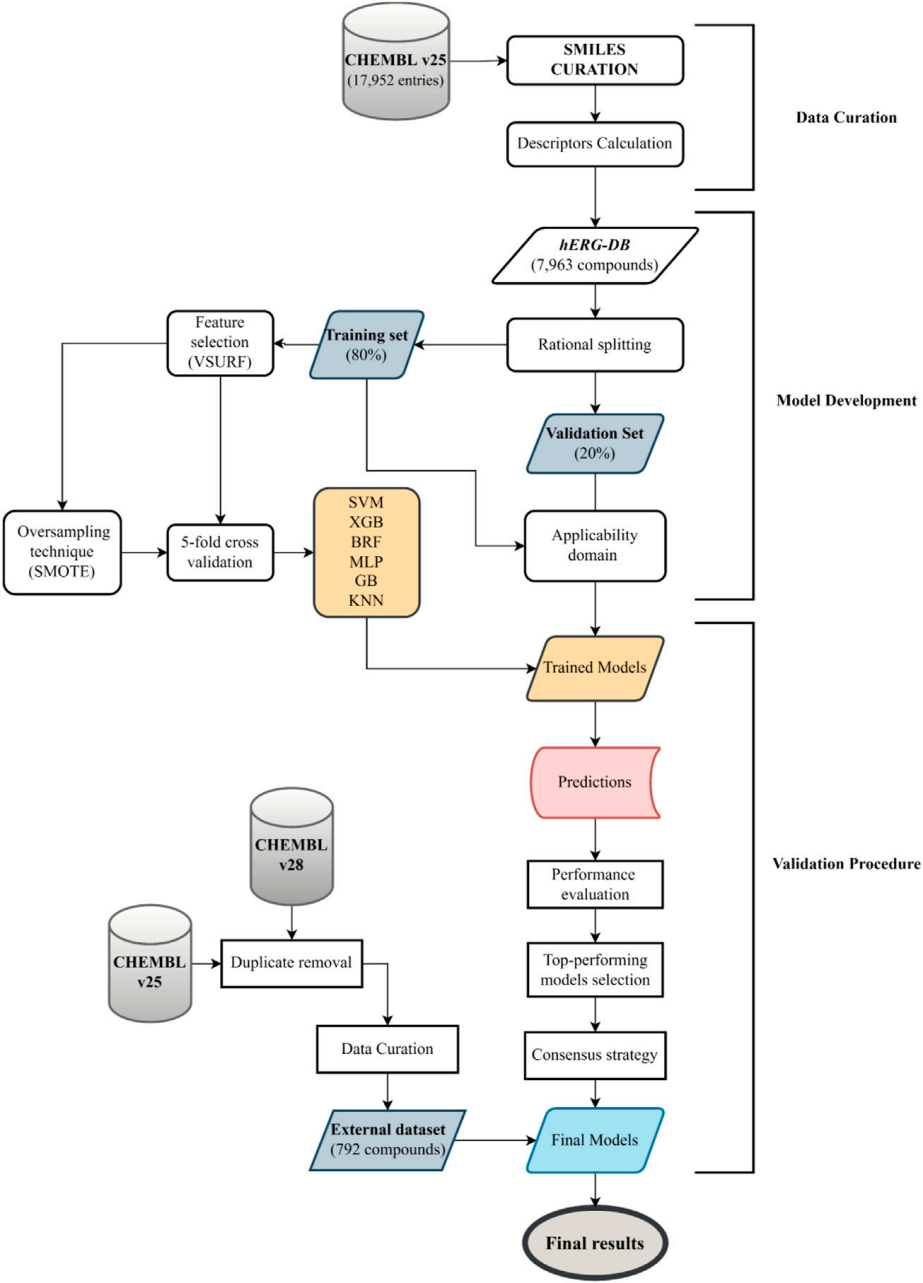
Flowchart showing the main steps of the adopted computational workflow.

In the present work, we developed QSAR models employing different ML algorithms, RF (Breiman, 2001), KNN (Altman, 1992), GB (Friedman, 2001), XGB (Chen and Guestrin, 2016), MLP (Haykin, 1994), and SVM (Vapnik, 1963) available in the KNIME Analytics Platform (v. 4.1.4) (Berthold et al., 2008). The dataset used to build the model consists of highly curated pIC_50_ values for 7,963 organic compounds. This dataset allows for the covering of a wide range of structural characteristics of hERG blockers and nonblockers and also a broad chemical space, as evident in Figure 1. Based on the literature, threshold values for the blocker/nonblocker classification vary from IC_50_ = 1 μM (pIC_50_ = 6) to IC_50_ = 10 μM (pIC_50_ = 5) (Jing et al., 2015; Li et al., 2017; Siramshetty et al., 2018, 2020; Choi et al., 2020). For this reason, we used these two thresholds to develop binary classification models. In addition, one set of models accounted for all of the descriptors generated by DRAGON v7.0.4 (Kode, 2017), while the other, only the pool of descriptors selected by VSURF (Genuer et al., 2010) (Supplementary Table S1). Notably, the performances returned by the two sets of the models are comparable, remarking the effectiveness of the selection variable strategy, as already experienced in previous works (Gadaleta et al., 2018b; Baderna et al., 2020; Lavado et al., 2020). Therefore, we have focused our attention on the results returned by the VSURF models, being less complex and easier to be implemented. However, the interested reader is referred to the supporting information for the performances in validation and 5-fold CV returned by all of the models trained with the entire set of descriptors (Supplementary Tables S3, S4). The discussion will focus on the most important metrics to determine the top-performing models, SE, SP, BA, and AUC, given the imbalance of TS. In addition, we used an ES as a temporal validation to assess the predictivity of the models in a real-life case study. Finally, for the sake of comparison, the performance of our top-selected models has been compared with that obtained on the ES with commonly employed and freely accessible models: DeepHIT (Ryu et al., 2020), CardioTox (Karim et al., 2021), Cardprep (Lee et al., 2019), ADMETlab (Xiong et al., 2021), and OCHEM consensus models I and II (Li et al., 2017). For the sake of clarity, Figure 2 shows the workflow that summarizes the main steps of the adopted computational protocol. Notably, the use of a rational approach for data split allowed us to minimize the risk of variation in performance due to different TS-VS divisions. Indeed, the iteration of the rational data-split procedure performed on models based on the entire set of descriptors does not show relevant differences in terms of statistical performance (Supplementary Table S5) with respect to models based on the TS-VS split presented here (Supplementary Table S4).

### Models developed using pIC_50_ = 6 as the toxicity threshold

Table 2 reports the computed performances on the VS returned by each model based on the activity threshold pIC_50_ = 6 without and with the application of the SMOTE (S) (Chawla et al., 2002). Performance refers only to chemicals included in the AD (see section Applicability Domain). Among the models trained on the unbalanced TS (nonblockers/blockers ratio equal to 5), the RF model, combined with a uniform size sampling strategy (BRF) to reduce the bias toward the majority class, returns the best performance. In particular, BRF is responsible for the most balanced statistics when predicting both positive and negative samples with a difference of SE (0.92) and SP (0.81) of only 0.11 and for the highest BA (0.87). All of the other classifiers are characterized by a high FN rate, despite returning acceptable values of BA. The gap between SP and SE ranges from 0.24 (SVM) to 0.40 in (MLP) and might be the result of the absence, in these models, of any procedure to properly consider the TS unbalance. The importance of having a balanced TS is supported by the statistics returned by the models taking advantage of the SMOTE. Indeed, some of them returned a significant performance improvement in predicting both positive and negative samples. (S)KNN and (S)SVM ensured the best performances among all of the developed models, with (S)SVM associated with the best BA (0.88), accounting for a SE of 0.91 and, as a consequence, for a low rate of false negatives. Building on these data, we can reasonably claim that the top-performing models to be selected for additional validation and consensus strategies are BRF, (S)KNN, and (S)SVM. This is supported also by the corresponding AUC values, being 0.95, 0.92, and 0.89, respectively. Remarkably, the 5-fold CV ensures the internal robustness of the three models with BRF, (S)KNN, and (S)SVM reaching BA as high as 0.79, 0.78, and 0.76, respectively (Supplementary Table S6).

**TABLE 2:** Performances on the VS of the models developed using pIC_50_ = 6 (top) and 5 (bottom). For each model, the following statistics are reported: balanced accuracy (BA), sensitivity (SE), specificity (SP), Matthews correlation coefficient (MCC), area under the ROC (AUC), number of true negatives (TNs), false positives (FPs), true positives (TPs), and false negatives (FNs). The top-performing model selected for additional validation is indicated in bold.

Toxicity threshold pIC_50_ = 6										
Balancing	Method	BA	SE	SP	MCC	AUC	TP	FP	TN	FN
-	**BRF**	**0.87**	**0.92**	**0.81**	**0.58**	**0.95**	**226**	**250**	**1088**	**19**
	GB	0.82	0.68	0.96	0.68	0.94	170	52	1286	75
	KNN	0.84	0.72	0.96	0.70	0.91	176	51	1287	69
	MLP	0.76	0.56	0.96	0.57	0.89	136	54	1284	109
	XGB	0.84	0.73	0.95	0.69	0.94	179	60	1278	66
	SVM	0.84	0.72	0.96	0.69	0.93	176	58	1280	69
SMOTE	(S)RF	0.85	0.73	0.96	0.72	0.95	178	46	1292	67
	(S)GB	0.83	0.72	0.95	0.66	0.82	177	72	1266	68
	**(S)KNN**	**0.86**	**0.86**	**0.85**	**0.59**	**0.92**	**214**	**209**	**1129**	**31**
	(S)MLP	0.81	0.86	0.76	0.48	0.91	211	322	1016	34
	(S)XGB	0.78	0.74	0.82	0.46	0.87	181	239	1099	64
	**(S)SVM**	**0.88**	**0.91**	**0.85**	**0.62**	**0.89**	**223**	**198**	**1140**	**22**
Toxicity threshold pIC_50_ = 5										
-	**BRF**	**0.83**	**0.85**	**0.82**	**0.67**	**0.92**	**655**	**147**	**663**	**114**
	**GB**	**0.83**	**0.84**	**0.82**	**0.66**	**0.92**	**646**	**147**	**663**	**123**
	KNN	0.83	0.84	0.82	0.66	0.91	647	151	659	122
	MLP	0.80	0.83	0.77	0.60	0.88	640	192	618	129
	XGB	0.83	0.84	0.82	0.65	0.91	643	150	660	126
	**SVM**	**0.83**	**0.86**	**0.80**	**0.66**	**0.91**	**659**	**163**	**647**	**110**
SMOTE	(S)RF	0.83	0.84	0.82	0.67	0.92	645	143	673	118
	(S)GB	0.83	0.84	0.82	0.64	0.92	637	147	672	123
	(S)KNN	0.82	0.86	0.78	0.64	0.90	652	178	641	108
	(S)MLP	0.77	0.84	0.69	0.65	0.85	645	143	665	126
	(S)XGB	0.84	0.85	0.83	0.68	0.90	681	136	647	115
	(S)SVM	0.84	0.84	0.84	0.68	0.91	650	130	678	121

Table 3 shows the performances on the VS of the three consensus models developed by integrating each possible pair of the three top-performing classifiers indicated above. Notice that all of the consensus models return very high BAs (0.91), thus outperforming all of the single classifiers. Importantly, irrespective of the single models involved in the consensus strategy, only a small fraction of compounds (<11%) has been excluded by the prediction because of a discordant classification. These results put forward the considered consensus strategies as being extremely powerful to maximize the predictive performance of the models developed when pIC_50_ = 6 is considered the toxicity threshold. These consensus models were further challenged via a successive temporal validation using the ES. Although the performances were worse than those obtained on the VS, as already experienced in previous works (Sheridan, 2013; Bosc et al., 2019) and similar to the performance observed with the 5-fold CV procedure, the consensus models are responsible for satisfactory values of BA and AUC. It is noteworthy that (S)KNN+(S)SVM (BA = 0.72; AUC = 0.73) and BRF+(S)KNN (BA = 0.72; AUC = 0.73) returned a more balanced statistic, thus outperforming BRF+(S)SVM (BA = 0.71; AUC = 0.72) (Table 3).

**TABLE 3:** Performance of the consensus models on the VS and on the ES (temporal validation) developed using pIC_50_ = 6 (top) and 5 (bottom). For each model, the following statistics are reported: balanced accuracy (BA), sensitivity (SE), specificity (SP), Matthews correlation coefficient (MCC), area under the ROC (AUC), number of true negatives (TNs), false positives (FPs), true positives (TPs), false negatives (FNs), and the total number of molecules (#). The top-performing models selected for temporal validation are indicated in bold.

Toxicity threshold pIC_50_ = 6											
Method	Dataset	BA	SE	SP	MCC	AUC	TP	FP	TN	FN	#
(S)SVM+(S)KNN	VS	0.91	0.93	0.90	0.72	0.93	207	117	1048	15	1387
	ES	0.72	0.66	0.77	0.34	0.73	55	107	365	29	556
BRF+(S)SVM	VS	0.91	0.95	0.87	0.69	0.95	215	153	1043	11	1422
	ES	0.71	0.60	0.81	0.33	0.72	53	102	454	35	644
BRF+(S)KNN	VS	0.91	0.94	0.88	0.68	0.95	208	152	1031	13	1404
	ES	0.72	0.68	0.76	0.33	0.73	53	112	348	25	538
Toxicity threshold pIC_50_ = 5										
**BRF + SVM**	**VS**	**0.87**	**0.89**	**0.86**	**0.75**	**0.93**	**619**	**108**	**608**	**74**	**1409**
	**ES**	**0.72**	**0.67**	**0.76**	**0.43**	**0.75**	**220**	**67**	**212**	**107**	**606**
BRF + GB	VS	0.87	0.88	0.86	0.74	0.93	618	104	622	86	1430
	ES	0.70	0.66	0.74	0.41	0.73	216	71	212	120	619
SVM + GB	VS	0.87	0.88	0.86	0.74	0.93	614	103	603	78	1398
	ES	0.71	0.66	0.75	0.41	0.74	223	69	213	116	621

### Models developed using pIC_50_ = 5 as toxicity threshold

Table 2 reports the performance returned by the validation performed on the VS for each model trained without using the SMOTE. Performance refers only to chemicals included in the AD (see section Applicability Domain). Similar BAs, ranging from 0.82 to 0.83, were obtained for all of the models, except for MLP responsible for the worst performance (BA = 0.80). Furthermore, all of the models return well-balanced statistics, with a difference between SP and SE ranging from 0.02 (XGB and GB) to 0.06 (MLP), consistent with the balanced composition of the TS. As expected, herein the SMOTE application did not lead to a significant performance improvement. Taken as a whole, these results suggest that, among all, the models developed using BRF, GB, and SVM as algorithms are the top-performing ones. This is evident looking at the computed BA and AUC values reported in Table 2. Importantly, the 5-fold CV confirms the internal robustness of these models (Supplementary Table S6) that were thus selected for further consensus and temporal validation procedures.

Again, the application of a consensus strategy led to a performance improvement in terms of both BA and AUC. In particular, Table 3 shows that all of the consensus models reach a BA equal to 0.87 and an AUC of 0.93, hence outperforming the BA_MAX_ (0.83) and AUC_MAX_ (0.92) obtained from the single models. Again, a small fraction of compounds (<11%) was unpredicted as a consequence of a discordant classification between the involved models. Ultimately, the performed temporal validation (Table 3) confirms the overall good predictivity of the consensus classifiers, with the BRF + SVM ensuring the best BA and AUC values (BA = 0.72, AUC = 0.75).

### Comparison with other classifiers available in the literature

**FIGURE 3:**
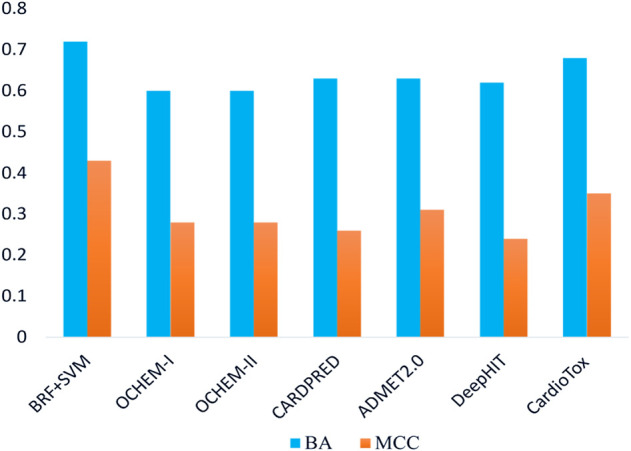
Comparison of balanced accuracies (BAs) and Matthews correlation coefficients (MCCs) for the selected model on the ES. Blue bars refer to BA, while orange bars refer to MCC.

The performances returned by the consensus models confirm that the integration of multiple strategies, applying a weight-of-evidence approach, leads to the detection and exclusion of erroneous predictions generated by the individual models, reinforcing, at the same time, those concordant. To the best of our knowledge, our consensus classifiers outperform on this VS (e.g., AUC_MAX_ = 0.95 and 0.93 when the toxicity threshold is pIC_50_ = 6 and 5, respectively) all of the binary classifiers of hERG-related cardiotoxicity available in the literature and built using the same toxicity thresholds. Encouraged by these pieces of evidence and aimed at performing a more detailed comparative analysis between our computational workflow and other ligand-based hERG-blockage predicting models available in the literature, the real-life application of six tools freely available and widely used for predicting the hERG blocking potential of chemicals, namely, Cardprep (Lee et al., 2019), ADMETlab (Xiong et al., 2021), OCHEM consensus models I and II (Li et al., 2017), DeepHIT (Ryu et al., 2020), and CardioTox (Karim et al., 2021) were challenged in temporal validation using the same ES herein employed. All of these tools are able to discern hERG blockers from nonblockers using as toxicity threshold pIC_50_ = 5 and, for this reason, their performances were compared with those returned by the best model (BRF + SVM) developed using the same threshold. Notice that our BRF + SVM model excluded ∼23% of compounds (∼5% being outside the AD and ∼18% as a consequence of discordant predictions) while OCHEM I and II discarded ∼14% compounds based on AD. Noteworthily, all of the other tools do not provide any AD-based filter to be applied. Furthermore, as the computation of AUC was not possible for all of the considered tools, being not able to provide a probability-based ranking, we used BA and MCC for the performance comparison. Remarkably, as shown in Table 4, the herein developed RF + SVM classifier showed more balanced statistics in predicting positive and negative samples than OCHEM I/II, ADMET 2.0, and DeepHIT models. In particular, OCHEM I/II presents a higher FN rate (SE = 0.24 and 0.38), whereas a higher FP rate characterizes ADMET 2.0 (SP = 0.36) and DeepHIT (SP = 0.44). As evident in Figure 3, BRF + SVM exhibits the highest BA and MCC (0.72 and 0.43, respectively) compared to all of the tested models, albeit excluding a fraction of compounds from the prediction, as previously mentioned. Hence, it can be reasonably considered as the best performing one in terms of real-life applicability.

**TABLE 4:** Comparison in terms of performance on the ES (temporal validation) of the best performing model presented in this study (BRF + SVM) and different classifiers available in the literature. The following statistics are reported: balanced accuracy (BA), sensitivity (SE), specificity (SPE), Matthews correlation coefficient (MCC), and the total number of molecules (#).

	BRF + SVM	OCHEM-I	OCHEM-II	Cardprep	ADMET2.0	DeepHIT	CardioTox
BA	0.72	0.60	0.60	0.63	0.63	0.62	0.68
SE	0.67	0.24	0.24	0.66	0.89	0.80	0.70
SP	0.76	0.95	0.95	0.59	0.36	0.44	0.65
MCC	0.43	0.28	0.28	0.26	0.31	0.24	0.35
**#**	**606**	**682**	**670**	**792**	**792**	**792**	**792**

### QSAR-hERG: A freely accessible KNIME workflow

**FIGURE 4:**
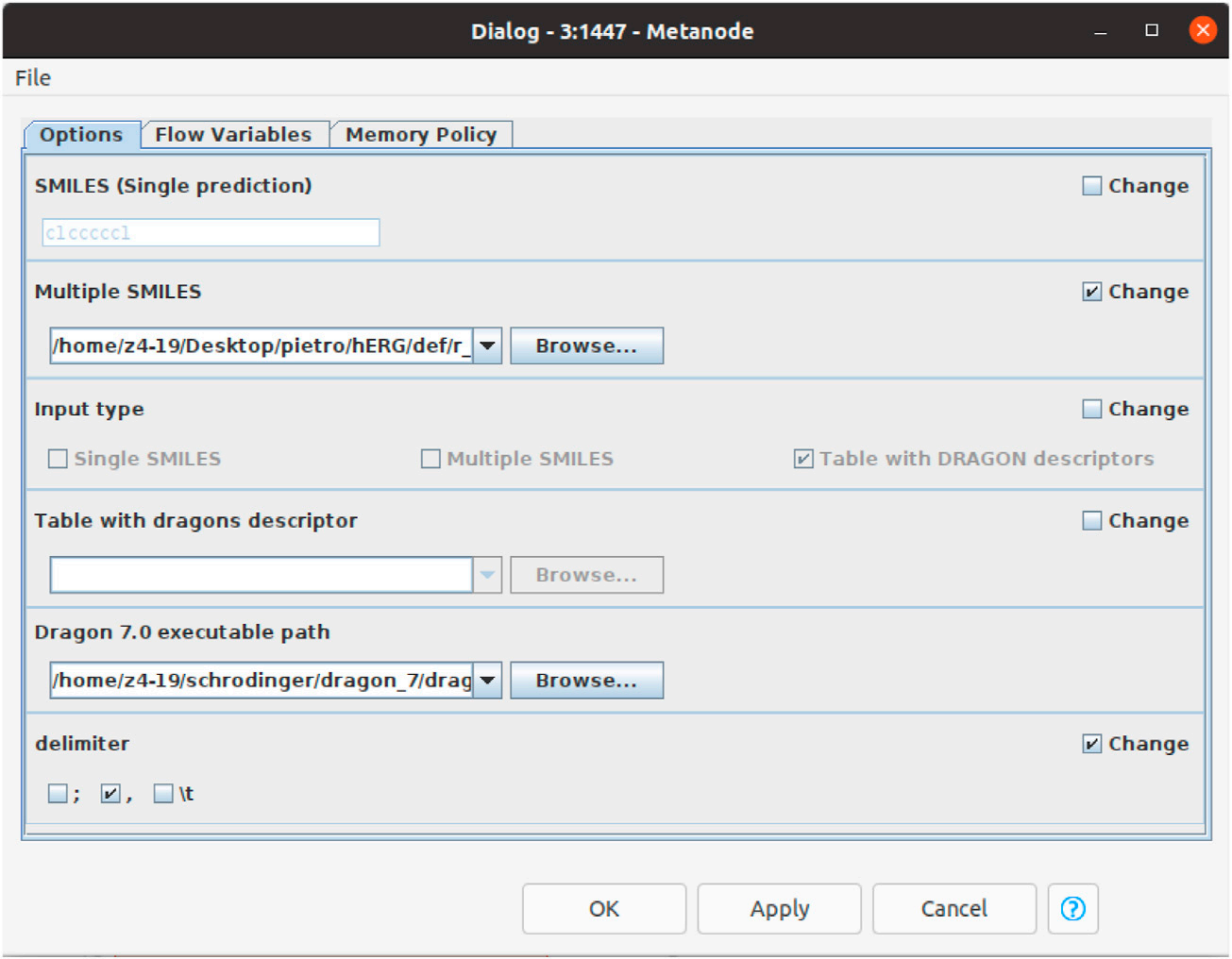
Dialog box to set up the calculation using the KNIME workflow.

**FIGURE 5:**
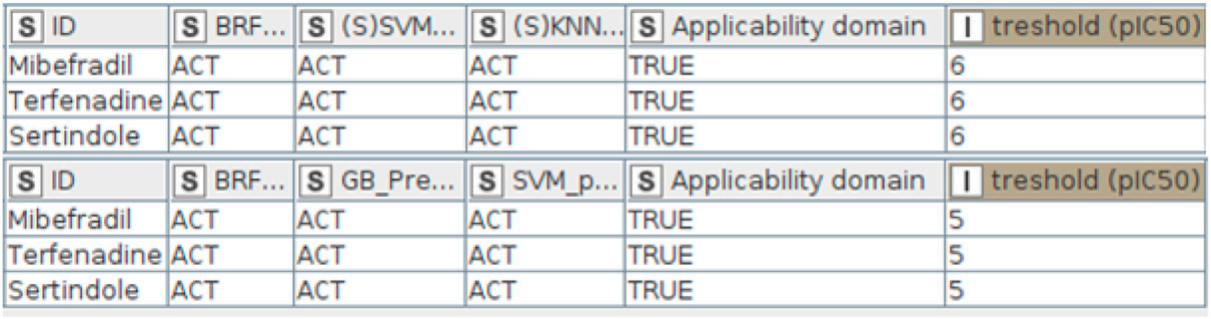
Output tables returned by the KNIME workflow for the three compounds examined: mibefradil, sertindole, and terfenafide.

The top-performing QSAR models are available as a KNIME workflow at https://github.com/PDelre93/hERG-QSAR. The implementation offers easy-to-use and intuitive options to use our predictive models. In the supporting information, a detailed guide explains how to install and perform fast hERG-related cardiotoxicity predictions. The graphical user interface (Figure 4) allows the users to choose the preferred way to proceed: 1) predict the activity of a single compound by manually entering the SMILES or 2) predict a batch of compounds from a SMILES list included in a. csv or. xlsx file. The workflow can automatically compute the required DRAGON descriptors (license is required). Alternatively, the user can include precalculated descriptors within the input file. The affinity toward the hERG channel is predicted using the top-performing models described above: BRF, (S)KNN, and (S)SVM for the activity thresholds pIC_50_ = 6; BRF, SVM, and GB for the activity threshold pIC_50_ = 5. At the end of the calculation, users can inspect the predictions generated by each model at pIC_50_ = 6 or 5, evaluating their reliability. Figure 5 shows the predictions returned by three compounds previously withdrawn from the market due to demonstrated hERG-related cardiotoxicity: mibefradil (Bezençon et al., 2017), sertindole (Sinha and Sen, 2011), and terfenadine (Sinha and Sen, 2011). Remarkably, all of the models predict the selected compounds as ACT (i.e., hERG-blockers). The column “applicability domain” expresses the reliability of the final prediction, which in our case is trustworthy (TRUE). It is worth noting that these findings agree with experimental data indicating pIC_50_ values of 6.24 for mibefradil (Bezençon et al., 2017), 7.83 for sertindole (Sinha and Sen, 2011), and 6.67 for terfenafide (Sinha and Sen, 2011).

## Conclusion

In this study, we developed 30 QSAR models based on 7,963 highly curated bioactivity data reported in ChEMBL (version 25) (Gaulton et al., 2012) and 1D and 2D descriptors computed by DRAGON 7.0.4 (Kode, 2017). By employing six machine learning algorithms, namely RF (Breiman, 2001), KNN (Altman, 1992), GB (Friedman, 2001), XGB (Chen and Guestrin, 2016), MLP (Haykin, 1994), and SVM (Vapnik, 1963), we implemented two sets of binary models differing for the considered toxicity threshold (pIC_50_ = 6 or pIC_50_ = 5). To maximize the performances, we followed three strategies for building ligand-based classifiers, namely: 1) VSURF (Genuer et al., 2010), to select relevant features to use in model construction, 2) the oversampling technique SMOTE (Chawla et al., 2002) to handle the unbalanced data; 3) a consensus approach to overcome single model limitations. Remarkably, the obtained results highlight the usefulness of these strategies, as testified by the high performances returned in the validation procedure. Importantly the performed temporal validation confirms the reliability of our models in real-life cases, given their ability to properly classify as hERG blockers or nonblocker compounds belonging to a repository (ChEMBL (Gaulton et al., 2012) v28) published after the data used for building TS and VS (ChEMBL (Gaulton et al., 2012) v25). Noteworthily, the models can be efficiently used in combination with structure-based strategies (Creanza et al., 2021) as testified by recent literature (Mansouri et al., 2016; Kamel et al., 2017). Finally, the performed comparative analysis indicates that the top-performing consensus model herein developed outperforms several commonly employed classifiers available in the literature. Our computational workflow is available to the cheminformatics community in the GitHub repository (https://github.com/PDelre93/hERG-QSAR), as valuable for a robust ligand-based prediction tool of hERG-related cardiotoxicity.

## Data Availability

The original contributions presented in the study are included in the article/Supplementary Material, and further inquiries can be directed to the corresponding authors.
