# Supplementary material for: Ligand-based prediction of hERG-mediated cardiotoxicity based on the integration of different machine learning techniques
Source: Front Pharmacol. 2022 Sep 5;13:951083. doi: 10.3389/fphar.2022.951083 (PMC9483173; doi:10.3389/fphar.2022.951083)
Supplement: Supplementary file 1 [file DataSheet4.PDF]

Ligand-based prediction of hERG-mediated cardiotoxicity based on the integration of different machine learning techniques

This workflow includes Quantitative Structure-Activity Relationship (QSAR) models to predict the hERG-related cardiotoxicity of chemicals.

Technical details

Data source

Data used for models development were extracted from the CHEMBL database (hiips://www.ebi.ac.uk/chembl/), keeping IC_{50} measures referring to hERG (target ID: CHEMBL240), obtained by assays conducted on human targets (“target_organism” = “Homo sapiens”) and marked as direct binding (“assay_type” = “B”). Two toxicity thresholds (pIC50 = 6 or pIC50 = 5) were used to develop two sets of binary classification models.

QSAR details

Six models are proposed, three for each threshold used, based on Dragon (v7.0.8) descriptors and different machine learning algorithms.

Threshold pIC_{50} = 6:

- Balanced random forest (**BRF**)
- k-Nearest Neighbors trained on data oversampled with SMOTE (**(S)KNN**)
- Support Vector Machine trained on data oversampled with SMOTE (**(S)SVM**)

Threshold pIC_{50} = 5:

- Balanced random forest (**BRF**)
- Gradient Boosting (**GB**)
- Support Vector Machine (**SVM**)

MODELS USAGE

Install the workflow

1) Download and install the KNIME Analytics Platform (hiips://www.knime.com/downloads/download-knime):
2) Go to “File -> Import KNIME Workflow”.
3) Tick “Select File:” and go to “Browse…”. Select the QSAR-hERG.knwf file and click to “Finish”.

The workflow now is in your “KNIME Explorer” menu (by default) on the left of the screen. Double click on the workflow to open it.

If some of the plugins used for the workflow are missing, a message will appear asking you to install the missing extensions. Click on “Ok”. The procedure will guide you in the installation of the missing extensions.

Generally, automatic installed plugins are:

- KNIME Base Chemistry Types & Node
- KNIME Chemistry Add-Ons
- KNIME Weka Data Mining Integration (3.7)
- Vernalis KNIME Nodes

The Enalos and DRAGON plugins may not be automatically identified.

To install Enalos Nodes for KNIME go to “File -> Preferences -> Install/update (doppio click) -> available software sites” and select on “KNIME Community Extensions (Experimental)”, then click on Apply and close. After that restart KNIME and follow the automatic installation procedure as before.

To install Dragon 7.0 descriptors node, drag and drop the file org.kode.dragon7_1.0.1.jar, available in this repository, in \”pathtoyourknimeinstallation”\knime_4.5.2\plugin.

Restart KNIME to activate the new plugins.

Run the workflow

By opening the dialog box with a double click on the Metanode, it is possible to choose the type of calculation to perform.

The user has three options:

a) predict the activity of a single compound by manually entering a SMILES.
b) predict the activity of a list of compounds by loading a CSV file. This file should include two columns: the first, named ‘ID’, which contains the identifiers, and the other, named ‘SMILES’, containing the SMILES list, one for each row.
c) Load a. CSV or .txt file that includes the identifier column ('ID') and the required descriptors in each column appropriately named.

Before starting the workflow, you need to specify the type of input loaded. Users can use options a) and b) only if a valid DRAGON license is available. To start the workflow, in this case, it is also necessary to specify the location of the DRAGON executable (For example, C:\Program Files (x86) \Dragon 7\dragon7shell.exe).

Now, Execute the workflow by clicking on the "Execute all the executable nodes" button on the top of the window or press Shift+F7.

How to read the output

The green light on the Metanode signals the correct execution of the process. By right-clicking on the element, users can select the output table of interest, with the predictions at a threshold equal to 6 or 5. The column header corresponds to the model used; ACT or INA represent the results. ACT indicates that one compound is predicted as an hERG-blocker, while INA is an hERG non-blocker. The column 'applicability domain' expresses the reliability of the final prediction, which should be trustworthy if the value is TRUE.
