# Supplementary material for: Ligand-based prediction of hERG-mediated cardiotoxicity based on the integration of different machine learning techniques
Source: Front Pharmacol. 2022 Sep 5;13:951083. doi: 10.3389/fphar.2022.951083 (PMC9483173; doi:10.3389/fphar.2022.951083)
Supplement: Supplementary file 3 [file DataSheet1.pdf]

Ligand-based prediction of hERG-mediated cardiotoxicity based on the integration of different machine learning techniques

Pietro Delre[1,2], Giovanna J. Lavado[3], Giuseppe Lamanna[1,2], Michele Saviano[1], Alessandra Roncaglioni[3], Emilio Benfenati[3], Giuseppe Felice Mangiatordi[1,*] and Domenico Gadaleta[3,*]

[1]CNR – Institute of Crystallography, Via Amendola 122/o, 70126 Bari, Italy

[2]Chemistry Department, University of Bari “Aldo Moro”, via E. Orabona, 4, I-70125 Bari, Italy.

[3]Laboratory of Environmental Chemistry and Toxicology, Department of Environmental Health Sciences, Istituto di Ricerche Farmacologiche Mario Negri IRCCS, Via Mario Negri 2, Milan, 20156, Italy

Supplementary material - Table of content

Table S1. The pool of descriptors selected by the R package VSURF for each pIC_{50} threshold.

Table S2. Optimized parameters for each algorithm.

Table S3.* 5-fold cross-validation performance of the models developed using pIC_{50} = 6 (top) and 5 (bottom) and the entire pool of descriptors computed by Dragon.

Table S4.* Performance on the VS of the models developed using pIC_{50} = 6 (top) and 5 (bottom) and the entire pool of descriptors computed by Dragon.

Table S5.* Average performance on the VS of the models developed using pIC_{50} = 6 (top) and 5 (bottom) and the entire pool of descriptors computed by Dragon. The performance was calculated by macro-averaging the statistical values resulting from 100 different TS-VS rational splits. Standard deviation (SD) associated to the average values was also reported. Notice that the performance is reported only for the best performing algorithms, as reported in the sections 4.1 and 4.2.

Table S6.* 5-fold cross-validation performance of the models developed using pIC_{50} = 6 (top) and pIC_{50} = 5 (bottom) and the pool of descriptors returned by the R package VSURF. The top-performing models selected for additional validation are indicated in bold.

***For each model, the following statistics are reported: Balanced Accuracy (BA), Sensitivity (SEN), Specificity (SPE), Matthews Correlation Coefficient (MCC), Area Under the ROC (AUC), number of True Negatives (TN), False Positives (FP), True Positives (TP) and False Negatives (FN).

Table S1.

pIC_{50} 6		pIC_{50} 5	
nDB	CATS2D_09_LL	nDB	P_VSA_ppp_con
nBnz	T(N..N)	nBnz	P_VSA_ppp_cyc
ARR	T(N..O)	Yindex	SpMax_AEA(bo)
MAXDN	T(N..F)	Chi_Dz(Z)	SpMax_AEA(dm)
MAXDP	T(O..O)	JGI3	SpMaxA_AEA(dm)
PCR	B01[N-O]	SpMax2_Bh(p)	Chi1_EA(dm)
Yindex	B02[O-O]	SpMin2_Bh(e)	SM09_EA(dm)
Chi_Dz(Z)	B06[N-O]	P_VSA_LogP_4	SM12_AEA(bo)
ATSC3m	F03[C-N]	P_VSA_p_2	SM15_AEA(bo)
ATSC7v	F03[N-O]	SpMaxA_EA(ed)	SM09_AEA(dm)
JGI3	F04[C-N]	nCb-	SM15_AEA(ri)
SpMax4_Bh(m)	F04[C-O]	C-006	Eig02_AEA(dm)
SpMax3_Bh(v)	F05[C-C]	O-058	nROH
SpMax5_Bh(v)	F05[N-O]	SdssC	C-008
SpMax8_Bh(v)	F05[O-O]	NssCH2	C-027
SpMax2_Bh(p)	F06[C-N]	NdssC	H-047
SpMin4_Bh(m)	F06[C-O]	CATS2D_00_LL	O-060
SpMin2_Bh(v)	F07[C-C]	CATS2D_03_LL	SsssN
SpMin2_Bh(e)	F07[C-O]	CATS2D_09_LL	CATS2D_09_DL
SpMin2_Bh(s)	F07[C-Cl]	T(N..N)	CATS2D_07_PL
P_VSA_LogP_4	F07[N-N]	T(N..O)	B04[N-O]
P_VSA_p_2	F08[C-N]	T(N..F)	B07[N-O]
SpMaxA_EA(ed)	F08[C-O]	T(O..O)	F01[C-C]
nCrs	F08[N-N]	F03[C-N]	F01[C-O]
nCb-	F10[C-C]	F04[C-O]	F01[N-N]
nRNR2	F10[N-N]	F05[C-C]	F02[C-N]
nArOR	MLOGP2	F05[N-O]	F02[N-N]
nArX		F06[C-N]	F03[C-O]
C-006		F06[C-O]	F07[C-N]
C-024		F07[C-C]	F09[C-N]
C-040		F07[C-O]	F09[C-O]
H-053		F08[C-N]	F09[C-Cl]
O-058		F08[C-O]	F10[C-F]
SssCH2		MLOGP2	PDI
SsssCH		RBN	
SdssC		RBF	
SaasC		D/Dtr06	
SssssC		DECC	
SaasN		X5v	
NssCH2		TI2_L	
NsssCH		ATSC8m	
NdssC		GATS4s	
CATS2D_04_DL		SpMin2_Bh(m)	
CATS2D_00_AA		SpMin3_Bh(m)	
CATS2D_02_AA		SpMin3_Bh(s)	
CATS2D_05_AA		SpMin4_Bh(s)	
CATS2D_06_AA		SpMin5_Bh(s)	
CATS2D_08_AA		P_VSA_m_3	
CATS2D_04_AL		P_VSA_v_3	
CATS2D_00_LL		P_VSA_ppp_L	
CATS2D_03_LL		P_VSA_ppp_D	
CATS2D_07_LL		P_VSA_ppp_A	

Table S2.

		Original set of descriptors				VSURF selection			
		pIC_{50}: 6		pIC_{50}: 5		pIC_{50}: 6		pIC_{50}: 5	
Model	optimized parameters	original TS	SMOTE TS	original TS	SMOTE TS	original TS	SMOTE TS	original TS	SMOTE TS
RF	number of trees	400	300	500	500	300	500	400	500
	equal size sampling	yes	no	yes	no	yes	no	yes	no
GB	number of trees	500	500	500	500	500	500	500	500
	learning rate	1	0.2	0.2	0.6	0.6	0.2	0.2	0.2
	Maximum tree depth	4	4	4	4	4	4	4	4
kNN	Number of neighbors to consider	5	5	3	3	3	5	5	7
	weight neighbors by distance?	yes	yes	yes	yes	yes	yes	yes	yes
MLP	Maximum number of iterations	100	100	100	100	1000	1000	1000	1000
	number of hidden layers	1	2	1	1	2	2	1	2
	number of hidden neurons per layer	4	2	8	10	12	2	12	10
XGB	eta	0.4	0.2	0.2	0.2	0.6	0.5	0.1	0.3
	gamma	0.1	0.4	0.2	0.2	0.1	0.2	0.5	0.2
	lamba	1	1	0.6	0.4	0.3	1	0.3	0.4
	alpha	0.8	0.8	0.4	0.8	1	0.5	0.1	0.3
	minimum child weight	7	4	1	8	4	1	4	1
SVM	cost	15.5		22.4		69.9	2	22.27	4
	gamma	3.6		1.8		0.3	0.34	1.78	0.7

Table S3.

Balancing	Method	BA	SE	SP	MCC	AUC	TP	FP	TN	FN
**Toxicity threshold pIC50 = 6**										
-	BRF	0.8	0.78	0.82	0.48	0.82	762	973	4415	221
	GB	0.7	0.45	0.96	0.49	0.77	440	205	5183	543
	KNN	0.72	0.51	0.94	0.48	0.88	504	349	5039	479
	MLP	0.7	0.47	0.93	0.42	0.95	464	400	4988	519
	XGB	0.71	0.45	0.96	0.5	0.86	442	189	5199	541
	SVM	0.74	0.54	0.94	0.51	0.9	530	326	5062	453
SMOTE	(S)RF	0.7	0.44	0.97	0.5	0.89	433	174	5214	550
	(S)GB	0.74	0.52	0.95	0.53	0.877	511	244	5144	472
	(S)KNN	0.77	0.78	0.75	0.41	0.81	769	1344	4044	214
	(S)MLP	0.74	0.67	0.81	0.39	0.809	656	1021	4367	327
	(S)XGB	0.7	0.43	0.97	0.5	0.858	425	160	5228	558
	(S)SVM	-	-	-	-	-	-	-	-	-
**Toxicity threshold pIC50 = 5**										
-	BRF	0.79	0.79	0.79	0.58	0.87	2444	694	2601	632
	GB	0.79	0.79	0.8	0.59	0.87	2421	652	2643	655
	KNN	0.74	0.76	0.73	0.49	0.81	2341	905	2390	735
	MLP	0.74	0.74	0.74	0.48	0.79	2270	843	2452	806
	XGB	0.79	0.78	0.8	0.58	0.87	2397	662	2633	679
	SVM	0.77	0.74	0.8	0.55	0.84	2279	649	2646	797
SMOTE	(S)RF	0.79	0.79	0.79	0.58	0.87	2444	694	2601	632
	(S)GB	0.79	0.79	0.8	0.59	0.87	2421	652	2643	655
	(S)KNN	0.74	0.76	0.73	0.49	0.81	2341	905	2390	735
	(S)MLP	0.74	0.74	0.74	0.48	0.79	2270	843	2452	806
	(S)XGB	0.79	0.78	0.8	0.58	0.87	2397	662	2633	679
	(S)SVM	-	-	-	-	-	-	-	-	-

Table S4.

Balancing	Method	BA	SE	SP	MCC	AUC	TP	FP	TN	FN
**Toxicity threshold pIC50 = 6**										
-	BRF	0.88	0.94	0.82	0.61	0.95	232	237	1109	14
	GB	0.8	0.62	0.98	0.69	0.93	153	25	1321	93
	KNN	0.83	0.71	0.94	0.65	0.91	175	77	1269	71
	MLP	0.79	0.65	0.93	0.57	0.9	159	94	1252	87
	XGB	0.82	0.67	0.97	0.69	0.93	166	44	1302	80
	SVM	0.84	0.74	0.94	0.67	0.92	181	75	1271	65
SMOTE	(S)RF	0.85	0.74	0.96	0.71	0.91	181	52	1294	65
	(S)GB	0.83	0.7	0.97	0.7	0.91	172	45	1301	74
	(S)KNN	0.87	0.93	0.81	0.58	0.9	228	251	1095	18
	(S)MLP	0.82	0.81	0.82	0.51	0.86	199	237	1109	47
	(S)XGB	0.82	0.76	0.88	0.55	0.91	186	168	1178	60
	(S)SVM	-	-	-	-	-	-	-	-	-
**Toxicity threshold pIC50 = 5**										
-	BRF	0.83	0.85	0.82	0.67	0.92	655	150	671	116
	GB	0.84	0.85	0.83	0.68	0.92	656	138	683	115
	KNN	0.81	0.85	0.77	0.63	0.9	656	185	636	115
	MLP	0.79	0.81	0.77	0.59	0.89	625	185	636	146
	XGB	0.84	0.85	0.82	0.68	0.91	659	146	675	112
	SVM	0.85	0.86	0.83	0.69	0.91	660	136	685	111
SMOTE	(S)RF	0.82	0.84	0.8	0.65	0.91	651	163	658	120
	(S)GB	0.84	0.84	0.83	0.67	0.92	649	141	680	122
	(S)KNN	0.81	0.86	0.77	0.63	0.91	662	192	629	109
	(S)MLP	0.77	0.85	0.69	0.55	0.86	659	254	567	112
	(S)XGB	0.84	0.86	0.83	0.69	0.9	663	140	681	108
	(S)SVM	-	-	-	-	-	-	-	-	-

Table S5.

	Toxicity threshold pIC50 = 6			
**Method**	**BA±SD**	**SE±SD**	**SP±SD**	**AUC±SD**
BRF	0.86±0.01	0.91±0.01	0.81±0.01	0.94±0.01
(S)KNN	0.85±0.01	0.90±0.01	0.80±0.01	0.91±0.01
**Toxicity threshold pIC50 = 5**				
BRF	0.84±0.01	0.85±0.01	0.83±0.01	0.92±0.01
SVM	0.82±0.01	0.83±0.01	0.81±0.01	0.90±0.01
GB	0.85±0.01	0.85±0.01	0.84±0.01	0.92±0.01

Table S6.

Balancing	Method	BA	SE	SP	MCC	AUC	TP	FP	TN	FN
**Toxicity threshold pIC50 = 6**										
-	**BRF**	**0.79**	**0.77**	**0.81**	**0.46**	**0.88**	**753**	**1032**	**4356**	**230**
	GB	0.71	0.46	0.96	0.5	0.87	449	201	5187	534
	KNN	0.72	0.5	0.94	0.49	0.84	496	308	5080	487
	MLP	0.66	0.36	0.96	0.42	0.8	355	191	5197	628
	XGB	0.71	0.47	0.96	0.5	0.88	458	213	5175	525
	SVM	0.71	0.49	0.93	0.45	0.84	482	374	5014	501
SMOTE	(S)RF	0.69	0.41	0.98	0.5	0.89	405	131	5257	578
	(S)GB	0.72	0.48	0.96	0.5	0.9	475	237	5151	508
	**(S)KNN**	**0.78**	**0.74**	**0.81**	**0.44**	**0.84**	**729**	**1032**	**4356**	**254**
	(S)MLP	0.73	0.68	0.78	0.37	0.83	670	1162	4226	313
	(S)XGB	0.7	0.44	0.96	0.48	0.88	437	211	5177	546
	**(S)SVM**	**0.76**	**0.67**	**0.85**	**0.45**	**0.85**	**658**	**785**	**4603**	**325**
**Toxicity threshold pIC50 = 5**										
-	**BRF**	**0.79**	**0.8**	**0.79**	**0.59**	**0.79**	**2458**	**698**	**2597**	**618**
	**GB**	**0.79**	**0.78**	**0.8**	**0.57**	**0.79**	**2392**	**658**	**2637**	**684**
	KNN	0.75	0.76	0.74	0.5	0.82	2344	852	2443	732
	MLP	0.73	0.72	0.74	0.46	0.78	2218	853	2442	858
	XGB	0.78	0.8	0.79	0.58	0.87	2398	668	2627	678
	**SVM**	**0.77**	**0.77**	**0.77**	**0.54**	**0.84**	**2369**	**762**	**2533**	**707**
SMOTE	(S)RF	0.79	0.72	0.75	0.58	0.87	2427	685	2610	649
	(S)GB	0.78	0.71	0.73	0.57	0.84	2392	658	2637	684
	(S)KNN	0.78	0.79	0.79	0.5	0.88	2407	917	2378	669
	(S)MLP	0.75	0.8	0.79	0.46	0.78	2310	946	2349	766
	(S)XGB	0.78	0.8	0.79	0.58	0.86	2397	645	2650	679
	(S)SVM	0.77	0.77	0.77	0.55	0.84	2425	767	2528	651
